# Identification of house price bubbles using robust methodology: evidence from Polish provincial capitals

**DOI:** 10.1007/s10901-021-09903-3

**Published:** 2021-09-28

**Authors:** Mateusz Tomal

**Affiliations:** Department of Real Estate and Investment Economics, Cracow University of Economics, Rakowicka 27, 31-510 Cracow, Poland

**Keywords:** Housing market, Speculative bubbles, Market fundamentals, Polish provincial capitals, IVX method, Poland, E31, R21, R31, R32

## Abstract

This article aims to check whether there has been a price bubble in the Polish major housing markets in recent years. To accomplish this goal, the log price-to-rent ratios in Polish provincial cities were analysed. In order to avoid incorrect conclusions, the log price-to-rent ratio using the instrumental variable estimation and ordinary least squares methods was decomposed into two components: fundamental and non-fundamental. The latter was then examined using the Phillips, Shi, and Yu procedure to detect explosive and downward movements. The results of the study showed that, in general, over 2011, actual log price-to-rent ratios in the analysed cities were below their fundamental values, i.e., a negative price bubble existed. However, more or less since the beginning of 2013, the surveyed markets have seen an increasing level of the non-fundamental component of the index under study, and its particularly explosive movements are visible in the first quarters of 2014. Finally, this analysis indicated future research directions and study implications for Polish policy-makers, housing investors, and households.

## Introduction

The real estate price bubble can be defined as an abnormal and self-sustaining price growth (Flood & Hodrick, 1990), unjustified by fundamental factors (Stiglitz, 1990), but rather due to misconceptions of investors expecting further property price increases (Case & Shiller, 2003). The 2007–2008 financial crisis has shown how important the issues of identifying price bubbles in the housing market are. Inaccurate detection of speculative behaviour in this market makes it impossible to take countermeasures what can lead in convection to the collapse of entire economies, and also has very negative consequences for the financial stability of households.

In recent years, also in Poland, experts and scientists have increasingly often analysed the dynamic growth of flat prices in the housing market, which both in the primary and secondary markets has averaged 50% over the last 14 years in real values (National Bank of Poland, 2020). It should therefore be asked whether this sustained and rapid growth is speculative or whether it is driven by housing market fundamentals, i.e., factors such as income, population growth or interest rates, changes which affect housing supply and demand and consequently the price level (Kholodilin et al., 2018; Maynou et al., 2021).^1^ However, scientific research to date has not provided an answer to this question, which is the result of a very small number of analyses of this type in the context of the Polish housing market.^2^ One such study was carried out by Brzezicka (2016), which examined the prices of flats in the city of Olsztyn and to what extent they are correlated with variables representing speculative behaviour. The results of this research showed that in 2007–2010 there was a price bubble in the housing market in the analysed city. In another study performed by Żelazowski (2018) the price-to-income ratio in Polish provincial cities was under investigation between 2003 and 2016. The author, using the generalised supremum augmented Dickey–Fuller (GSADF) test, detected that the residential market in Poland before the last great financial crisis also experienced the phenomenon of price bubbles. These conclusions were confirmed for the city of Warsaw by Krušinskas (2012), who also examined the price-to-income indicator. In turn, the latest research on the identification of price bubbles in the Polish residential market was performed by Czerniak and Kawalec (2020). The authors used both methods based solely on housing prices, such as the Bordo and Jeanne index (2002) as well as methods based on price-to-rent and price-to-income ratios to assess this phenomenon. The above study has shown that in recent years there has been no speculative bubble in the Polish real estate market, but rather favourable conditions for the growth of irrational behaviour.

As mentioned earlier, the analysis of housing market bubbles is based both on the prices themselves (see, e.g., Kibunyi et al., 2017; Chen & Chiang, 2020; Coskun et al., 2020) but also on relational indicators, in particular the price-to-rent ratio (see, e.g., Mikhed & Zemcík, 2009; Yiu et al., 2013; Greenaway-McGrevy & Phillips, 2016; Zhang et al., 2017; Li et al., 2019; Clark & Lomax, 2020) or a price-to-income ratio (see, e.g., Fraser et al., 2008; Chen & Cheng, 2017; McMillan & Speight, 2010) in the absence of rental data. The use of the latter indicators is preferable because both housing prices and rents can exhibit explosive behaviour over the same period, making it difficult to conclude on the existence of a bubble when based solely on prices. In the context of the price-to-rent ratio, as Shi et al. (2016) aptly notes, in the absence of a bubble, housing prices and rents should share the same trend in the long term, which is due to the treatment of house as a supplier of housing goods. However, very often the value, and consequently the price, of a dwelling is not only related to the goods it provides, but also to other factors, including those of a speculative nature, which cause prices to deviate significantly from their market fundamentals, leading to a bubble.

It should also be stressed that the detection of price bubbles in the housing market solely on the basis of prices or the relational indicators cited above is insufficient. This is because this type of study does not provide information on whether a potential price increase is justified, in other words, whether it is driven by market fundamentals. This problem has been recognised in recent years by researchers who have developed new methods of analysing housing market bubbles. In this respect, the simplest method of identifying them in the market in question is to compare the actually observed data with those predicted by an econometric model which takes into account the predictors explaining housing supply and demand. This type of analysis was conducted by Hui and Yue (2006) for the cities of Beijing and Shanghai. In addition, scholars analysed the relationship between housing prices, their fundamental and non-fundamental values in a more formal way, i.e., by applying the unit root and co-integration tests. For example, Campbell and Shiller (1987) proposed a methodology for bubble detection that relies on testing the stationarity of actual and fundamental prices.^3^ In particular, when both series are nonstationary or when the actual price is nonstationary, and its fundamental counterpart is, then a bubble can be said to exist. However, the unit root and co-integration tests cannot detect explosive bubbles when they periodically collapse (Evans, 1991). Consequently, investigators have proposed several alternative methods to address this limitation. Among others, Phillips et al. (2011) suggested using the recursive implementation of a right-tail unit root test and a supreme test. The drawback of the above method lies in the fact that it is designed to detect single bubble episodes in a time series. Therefore, Phillips et al. (2015a, b) constructed an alternative procedure (PSY) that differs from the previous one by being more flexible, i.e., when repeatedly using a right-tail unit root test, both the start and endpoints of the subsample are changed. The PSY method was successfully used by Shi et al. (2016) to analyse the Australian housing market and then by Shi (2017) for regional housing markets in the US, but in the latter case, VAR modelling was used to decompose the log price-to-rent ratio into fundamental and non-fundamental components before applying the PSY test. Then, Shi (2017) checked whether the non-fundamental component is characterised by explosive movements or just a random walk process employing the PSY procedure. However, as indicated by Shi and Phillips (2020), the implementation of the VAR model to decompose the log price-to-rent ratio is problematic and causes the accumulation of errors while analysis. Therefore, in order to extract the fundamental and non-fundamental values of the examined index, the above authors proposed the use of the instrumental variable estimation (IVX) method instead of VAR modelling, which increases the accuracy of the entire procedure, is easy to use and also allows the consideration of nonstationary time series in the study.

When taking into account the above literature review on the identification of speculative behaviour in the housing market, it should be concluded that there is still a large research gap in the context of the residential market in Poland. In particular, it should be emphasised that there is no single position in the available literature that would analyse the phenomenon of price bubbles in the Polish housing market while considering the dynamics of housing market fundamentals. A small number of studies in other countries using advanced statistical methods should also be highlighted, as Shi et al. (2016) also points out. Therefore, this article aims to fill the identified gap and check whether there has been a price bubble in the Polish major housing markets in recent years, using a novel approach proposed by Shi and Phillips (2020) that allows decomposing the log price-to-rent ratio into fundamental and non-fundamental components in a very accurate way. This study not only makes a major contribution to the current literature on the subject but will also serve as a basis for cutting speculation by experts and policy-makers who do not agree on the nature of the dynamic increase in housing prices in Poland, which will increase the rationality of all Polish housing market players.

The rest of the articles are organised as follows. Section 2 presents a theoretical way of decomposing the log price-to-rent ratio into fundamental and non-fundamental components, whereas the characteristics of the Polish housing market is provided in Sect. 3. The next Sect. 4, presents data used for research and methods of decomposition of the log price-to-rent ratio as well as bubble detection in the housing market. In turn, Sect. 5 section includes the results of the research and their discussion. The last Sect. 6 deals with the main findings of the conducted study as well as their limitations, implications and future research directions.

## Housing bubble theoretical framework

The starting point for the theoretical consideration of the housing bubble is the one-period gross return to housing given by the formula (Campbell et al., 2009; Liu et al., 2017):

1 $${g}_{t+1}=\frac{{p}_{t+1}+{r}_{t+1}}{{p}_{t}}$$

where ${p}_{t}$ (${r}_{t}$) is the real housing price (rent) at time $t$. Equation (1) can also be presented after logging as:

2 $$\mathrm{log}{g}_{t+1}=\mathrm{log}\left(\frac{{p}_{t+1}+{r}_{t+1}}{{p}_{t}}\right)=\mathrm{log}({e}^{\mathrm{log}{p}_{t+1}}+{e}^{\mathrm{log}{r}_{t+1}})-\mathrm{log}{p}_{t}$$

Then, using the Tylor approximation for Eq. (2), the following expression is obtained for the log price-to-rent ratio (Shi & Phillips, 2020):

3 $$\mathrm{log}{p}_{t}-\mathrm{log}{r}_{t}=\mu +\sigma \left(\mathrm{log}{p}_{t+1}-\mathrm{log}{r}_{t+1}\right)+\Delta \mathrm{log}{r}_{t+1}-{\mathrm{log}g}_{t+1}$$

where $\mu =-\mathrm{log}\sigma +\left(1-\sigma \right)\left(\overline{\mathrm{log} {p }_{t}}-\overline{\mathrm{log} {r }_{t}}\right)$, $\sigma =\frac{{e}^{\overline{\mathrm{log} {p }_{t}}}}{\left({e}^{\overline{\mathrm{log} {p }_{t}}}+{e}^{\overline{\mathrm{log} {r }_{t}}}\right)}$. In the next step, by iterating forward of Eq. (3) the log price-to-rent ratio can be decomposed into a fundamental (${f}_{t}$) and a bubble/residual component (${b}_{t}$):

4 $$\mathrm{log}{p}_{t}-\mathrm{log}{r}_{t}={f}_{t}+{b}_{t}$$

where

5 $${f}_{t}=\frac{\mu }{1-\sigma }+\sum_{j=0}^{\infty }{\sigma }^{j}(\Delta \mathrm{log}{r}_{t+1+j}-{\mathrm{log}g}_{t+1+j})$$

6 $${b}_{t}=\underset{k\to \infty }{\mathrm{lim}}{\sigma }^{k}\mathrm{log}{p}_{t+k}$$

On the basis of Eq. (5), it should be concluded that the value of the fundamental component in period $t$ depends on future rent rates of change ($\Delta \mathrm{log}{r}_{t+1+j}$) and future logarithmic gross returns to housing (${\mathrm{log}g}_{t+1+j}$). The latter can be presented as the sum of the real interest rate ${i}_{t}$ and the fixed risk premium $\vartheta$ (Shi, 2017), i.e., ${\mathrm{log}g}_{t+1}=\vartheta +{i}_{t+1}+{\varepsilon }_{t+1}$, where ${\varepsilon }_{t}$ is the error term with a zero mean. By inserting the above presented decomposition of ${\mathrm{log}g}_{t+1}$ to Eq. (5) we obtain a formula for the fundamental component, which is a function of future rent growth rates $\Delta \mathrm{log}{r}_{t+1+j}$ and future real interest rates ${i}_{t+1+j}$:

7 $${f}_{t}=\frac{\mu -\vartheta }{1-\sigma }+\sum_{j=0}^{\infty }{\sigma }^{j}(\Delta \mathrm{log}{r}_{t+1+j}-{i}_{t+1+j}-{\varepsilon }_{t+1+j})$$

The estimation of Eq. (7) is carried out in 3 three steps:

1. Predicting future stream of $\Delta \mathrm{log}{r}_{t+1+j}$ and ${i}_{t+1+j}$ using for example VAR modelling. The construction of a VAR model also should take into account other variables that influence residential demand through the real rent and real interest rate.
2. Calibration of unknown parameters $\mu$, $\vartheta$, $\sigma$ based on whole test sample data.
3. Calculation of the fundamental component of the log price-to-rent ratio at time $t$ by combining the results obtained in steps 1 and 2 using Eq. (7).

Coming back to the analysis of the residual component ${b}_{t}$, it should be stressed that in case of the normal behaviour of the housing market this component goes to zero (Caspi, 2016), i.e., $\lim_{k \to \infty } \sigma^{k} \log p_{t + k} = 0$. However, in the event of a price bubble, the limit of the above function is different from zero and the ${b}_{t}$ component is characterised by explosive behaviour such that (Diba & Grossman, 1988):

8 $${{E}_{t}(b}_{t+1})=\frac{1}{\sigma }{b}_{t}$$

where $\frac{1}{\sigma }>1$, ${E}_{t}$ is the operator of conditional expectations.

## The characteristics of the major housing markets in Poland

**Fig. 1:**
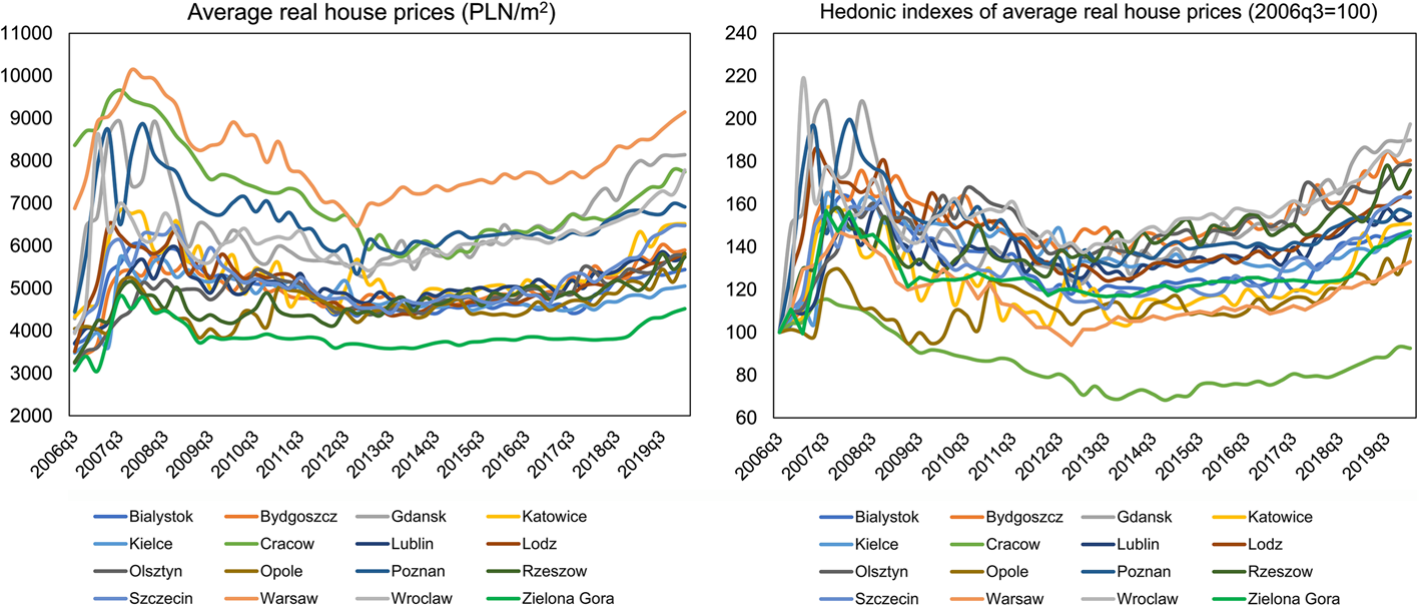
Real average house prices in Polish provincial capitals in the period 2006q3–2020q1. *Note* The data refers to the primary flat market

**Fig. 2:**
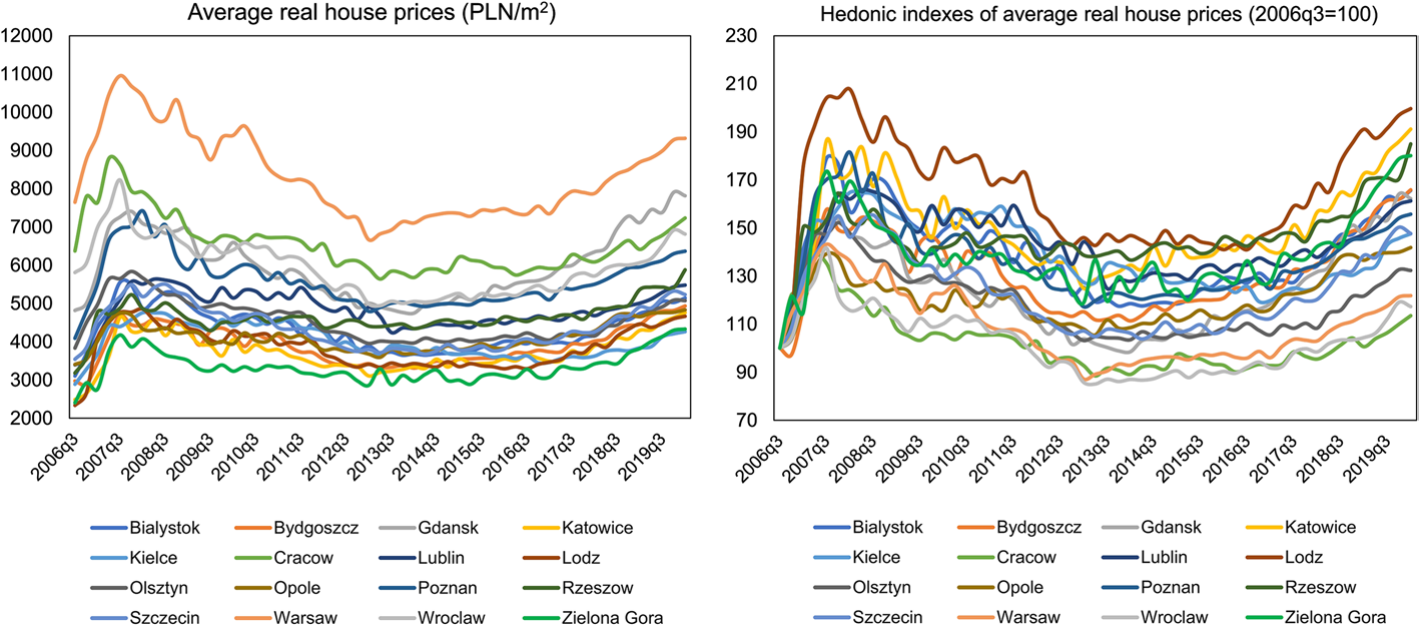
Real average house prices in Polish provincial capitals in the period 2006q3–2020q1. *Note* The data refers to the secondary flat market

The residential market in Poland has been developing very dynamically for several years. This is mainly due to the local housing markets operating in the capital cities of Polish voivodeships. Figures 1 and 2 present the changes in real residential prices in the primary and secondary market, respectively. For the last 14 years, by far the highest prices in real terms in both markets, approaching the level of 10,000 Polish Zloty (PLN) per 1 m^2^, have been observed in Poland's capital city, Warsaw. Cracow, Gdansk, Poznan and Wroclaw form a group of cities where prices range between PLN 6000–8000 per 1 m^2^. Much lower prices are noted in smaller cities such as Lublin, Kielce and Rzeszow. In the case of the price change dynamics, the situation is opposite, i.e., smaller residential markets are characterised by higher growth rates. The leaders in this respect are Wroclaw and Lodz, where prices between 2006q3 and 2020q1 increased by over 95% in the primary and secondary market, respectively. Overall, in both markets, prices rose on average by more than 50% when considering all surveyed cities.

**Fig. 3:**
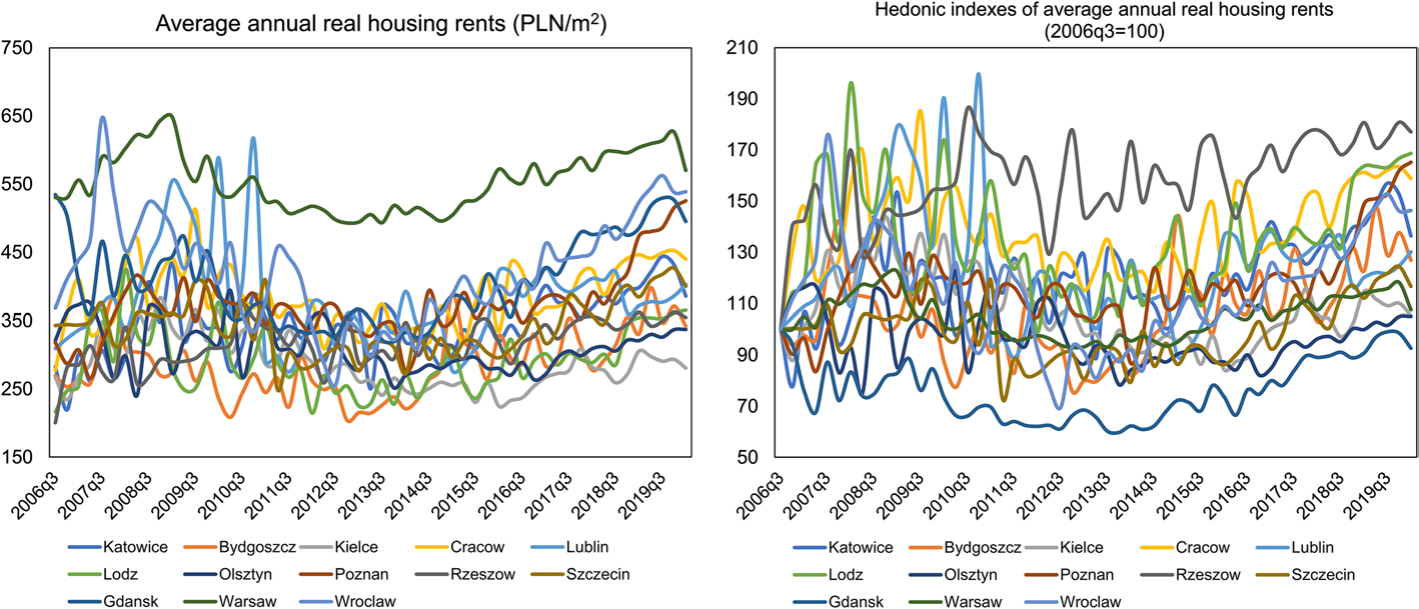
Real annual average housing rents in Polish provincial capitals in the period 2006q3–2020q1. *Note* The data refers to the flat residential market. Due to a lack of data, it is impossible to separate rental prices in the primary and secondary markets. Moreover, data for Opole, Zielona Gora and Bialystok are not presented because information on rent levels for the entire analysed period was missing

In the case of residential rents, it can be seen that, compared to sales prices, their volatility over time is much greater, as shown in Fig. 3. Throughout the analysed period, by far, the highest average annual transaction rents were in Warsaw (approx. PLN 550 per 1 m^2^). Similarly, to sales prices, high rents can also be found in Cracow, Gdansk, Poznan and Wroclaw, while the lowest in Kielce, which is one of the smallest Polish provincial cities in terms of population. It is also a distinctive feature of the dynamics of the Polish housing market that both sales prices and rents in major Polish cities do not converge to a single equilibrium but form convergence clubs, as can be seen in Figs. 1, 2, and 3 and has also been demonstrated empirically by Tomal (2019a, 2021a, b).

## Methodology

The study procedure is based on the new approach outlined by Shi and Phillips (2020), which simultaneously is able to detect bubble and downward movements of analysed time series. Furthermore, some adjustments have been made to the above-mentioned approach, highlighted in the subsequent sections. Specifically, after defining the study area and data used for the empirical survey, this research for each analysed city follows three main steps:

1. Decomposing the log price-to-rent ratio into fundamental and non-fundamental components using the multivariate triangular predictive model estimated by the IVX method.
2. Detection of the bubble and downward movements in the non-fundamental component of log price-to-rent ratio using the PSY procedure.
3. Implementation of a robustness test.

### Study area

**Fig. 4:**
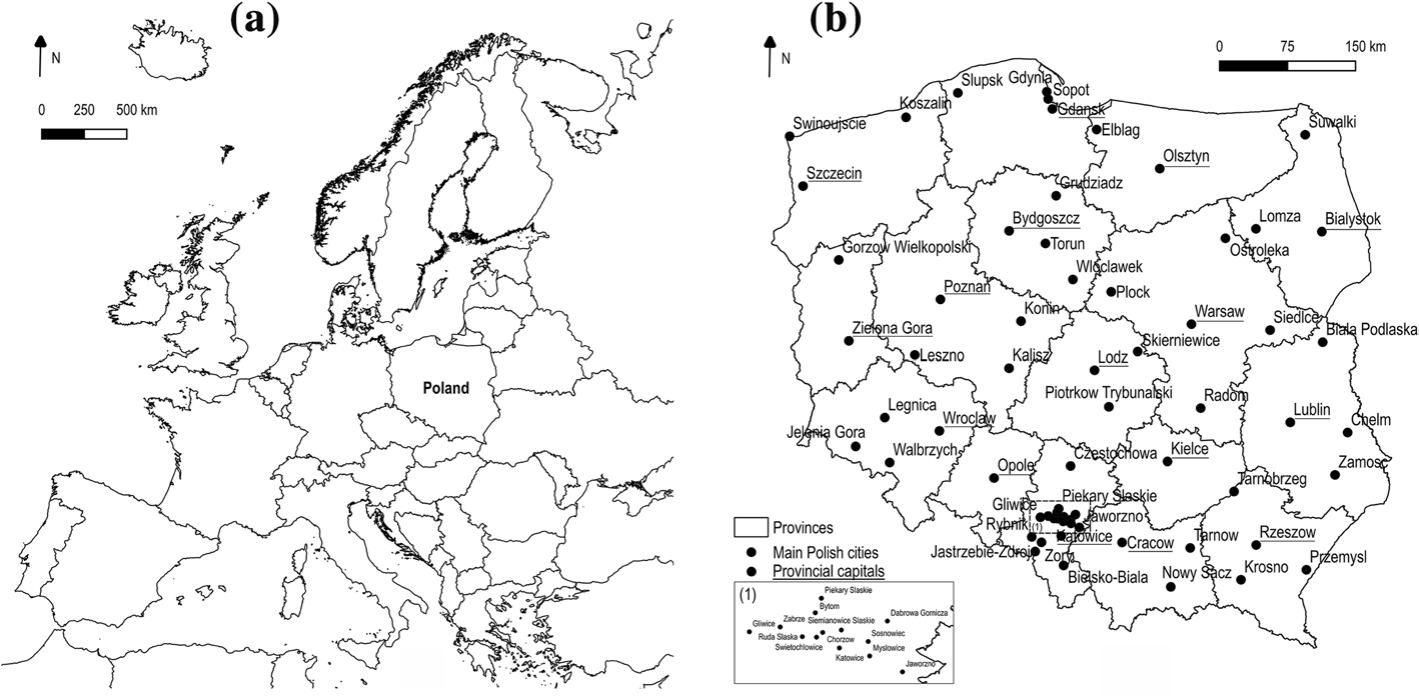
**a** Poland's location in Europe and **b** the division of Poland into provinces with the designation of their capitals

The study area includes local residential markets operating in the capital cities of Polish voivodeships, the location of which is shown in Fig. 4a, b. The choice of Poland as the place of analysis, and subsequently the major cities located within its territory, is not accidental. First, as Tomal (2021a, b) notes, the housing market in Poland is under-researched. Second, the knowledge of bubble and downward behaviours in Poland is important not only for Polish stakeholders but also for foreign entities, which results from spillovers between real estate markets in different countries (Agyemang et al., 2021). Third, the Polish housing market has long suffered from a shortage of sufficient housing units, resulting in increased activity of residential developers and subsequent price growth of properties in both the primary and secondary residential markets. In this context, awareness of the existence of price bubbles in the housing market is crucial for providers of new housing units as well as local and central legislators. In the price bubble period, the former should limit the scale of new investments and freeze the price level, while the latter, for example, can cool down the market through the increase of interest rates. Fourth, the Polish residential rental market has started to become a place of institutional investments for several years. Therefore, the lack of reliable information on the functioning of the housing market in Poland, also in terms of the presence or absence of price bubbles, may discourage potential foreign investors. In the end, the research encompassed housing markets in Polish voivodeship cities, which will enable a more detailed analysis of the bubble phenomenon than in the case of a nationwide study.

### Data

The main indicator used to identify speculative behaviour in the Polish residential market in this study is the log price-to-rent ratio calculated as a logarithmic quotient of the real average selling price per 1 m^2^ of flats from the secondary market (${p}_{t}$) and the real annual average rent per 1 m^2^ (${r}_{t}$) of flats. Data on the level of housing rents and prices were obtained from the National Bank of Poland (NBP). For the construction of price-to-rent ratios, sales prices from the secondary rather than the primary market were used since the vast majority of flats for rent come from the former market.^4^ Moreover, flats for sale from the primary market in Poland are almost always unfinished, in contrast to the flats offered for rent.

Then following Czerniak and Kawalec (2020), prices and rents were converted into real values through deflation with a quarterly HICP (Harmonised Index of Consumer Prices) reported for Poland. The data collected are quarterly and cover the period 2006q3–2020q1.^5^ Unfortunately, due to the deficiencies in the NBP database, it was not possible to obtain a longer time series. Because of data gaps, it was also unable to analyse three (Bialystok, Zielona Gora, Opole) of the sixteen Polish regional capitals. Moreover, no quarterly adjustment has been made to the time series used in the study due to the fact that this operation may cause a change in the time properties of the analysed variables (Montañés & Olmos, 2013) and, in particular, makes the ADF test less powerful (Ghysels & Perron, 1993), which is the basis of the PSY procedure.

**Fig. 5:**
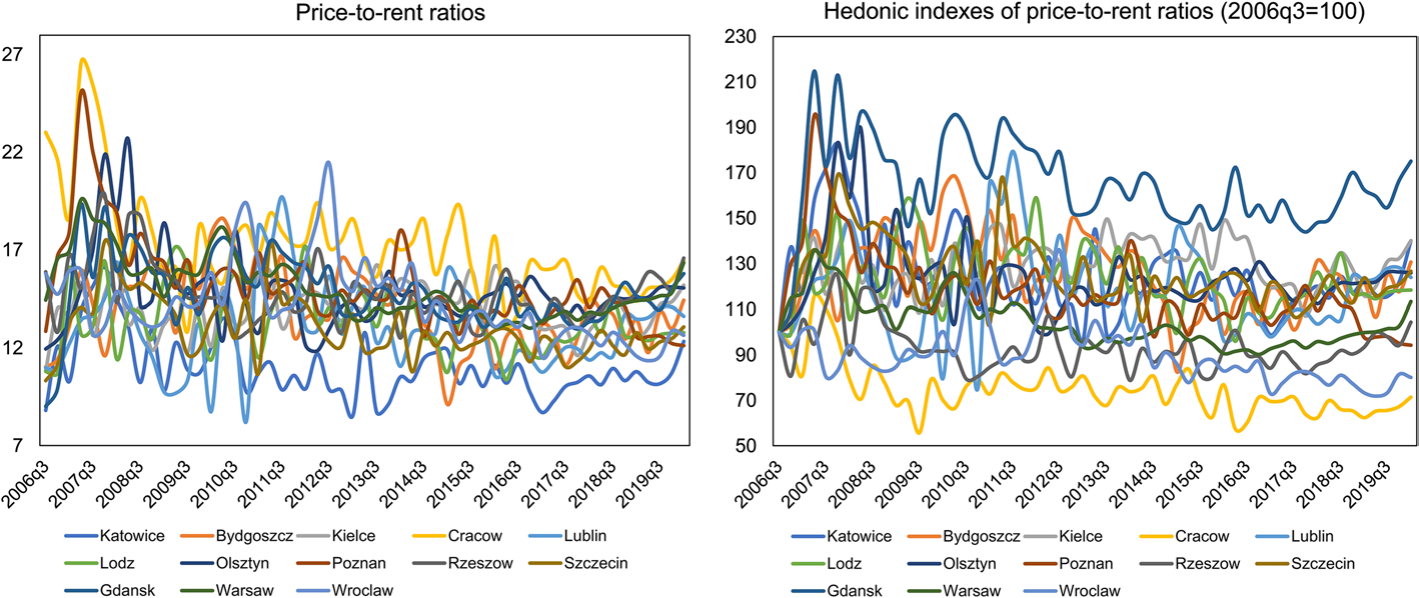
Price-to-rent ratios in Polish provincial capitals in the period 2006q3–2020q1

As shown in Fig. 5, the price-to-rent ratios in Polish cities over the last 14 years have been behaving quite variably, oscillating around 7–27. In particular, during the 2007–2008 financial crisis, the highest values of the price-to-rent ratio can be observed, which then started to strongly decrease. Since 2010 or so, a stabilisation of the analysed ratio has been noticed. Studying the price-to-rent ratios between cities, one should point out that renting is most profitable for households in Cracow, while the least in Katowice where the purchase of a house is recommended due to very low values of the examined ratio in the whole period under research. What is also important, the biggest increase of the price-to-rent ratio between 2006q3 and 2020q1 (over 75%) is present in Gdansk, and the smallest in Cracow (decrease by 29%).

This study also used data on fundamental factors that affect housing demand and supply. These variables are necessary to calculate the fundamental component of the log price-to-rent ratio. The housing market fundamentals were selected based on previous research and specifically describe: log new housing stock (Shi & Phillips, 2020; Shi et al., 2020), log real monthly gross salary (Shi, 2017; Shi et al., 2020), log number of unemployed (Adams & Füss, 2010; Shi, 2017; Shi et al., 2020), real interest rate (Adams & Füss, 2010; Shi et al., 2020), log real money supply (Adams & Füss, 2010), log real new household borrowing (Oikarinen, 2009), dynamic consumption index (Adams & Füss, 2010), and log real annual average log rent (Shi & Phillips, 2020; Shi et al., 2020). Table 1 provides details on how the data were obtained and the specifics of each variable.

**Table 1:** Detailed information on data used in the study

Variable	Institution	Access address	Unit	Level of availability^a^	Additional information
*Prices in the housing market*					
Log real average selling price	National Bank of Poland	https://www.nbp.pl/home.aspx?f=/publikacje/rynek_nieruchomosci/index1.html	PLN/m^2^	Local	Real values obtained by deflation with a quarterly HICP
Log real annual average log rent	National Bank of Poland	https://www.nbp.pl/home.aspx?f=/publikacje/rynek_nieruchomosci/index1.html	PLN/m^2^	Local	Real values obtained by deflation with a quarterly HICP
*Deflator*					
HICP	Central Statistical Office in Poland	https://stat.gov.pl/en/topics/prices-trade/price-indices/harmonized-indices-of-consumer-prices-hicp,15,1.html	Number	National	The reference year is 2015
*Housing market fundamentals*					
Log new housing stock	Local Data Bank of the Central Statistical Office in Poland	https://bdl.stat.gov.pl/BDL/dane/podgrup/temat	Unit	Local	NA
Log real monthly gross salary	Local Data Bank of the Central Statistical Office in Poland	https://bdl.stat.gov.pl/BDL/dane/podgrup/temat	PLN	Regional	Real values obtained by deflation with a quarterly HICP
Log number of unemployed	Local Data Bank of the Central Statistical Office in Poland	https://bdl.stat.gov.pl/BDL/dane/podgrup/temat	Person	Local	NA
Real interest rate	National Bank of Poland	https://www.nbp.pl/home.aspx?f=/dzienne/stopy_archiwum.htm: NBP reference rate; https://www.nbp.pl/home.aspx?f=/statystyka/bazowa/bazowa.htm: inflation	%	National	Calculated as NBP reference rate minus inflation
Log real money supply (M3)	National Bank of Poland	https://www.nbp.pl/home.aspx?f=/statystyka/pieniezna_i_bankowa/m3.html	PLN	National	Real values obtained by deflation with a quarterly HICP
Log real new household borrowing	Polish Bank Association	https://www.amron.pl/strona.php?tytul=raporty-amron-sarfin	PLN	Local	Real values obtained by deflation with a quarterly HICP
Dynamic consumption index	Polityka Insight	http://www.indekskonsumpcji.pl/	%	National	The index represents real dynamics of consumption in Poland

^a^Local level means that data are available for the area of the city. The regional level means that data are available for the province in which the city is located. The national level means that data are available for the country, in this case, Poland. NA means not applicable

### Log price-to-rent ratio decomposition method

It should be noted that the calculation of the fundamental component of the log price-to-rent ratio using Eq. (7) requires a multi-stage procedure, causing the accumulation of errors in each subsequent step (Shi & Phillips, 2020). Therefore, the decomposition of the log price-to-rent ratio has been made using the new approach outlined by Shi and Phillips (2020) enabling to estimate the fundamental component almost directly. This approach is based on modelling the growth rate of the price-to-rent ratio (${y}_{t}=\Delta \mathrm{log}{p}_{t}-\Delta \mathrm{log}{r}_{t}=\mathrm{log}\left(\frac{\frac{{p}_{t}}{{r}_{t}}}{\frac{{p}_{t-1}}{{r}_{t-1}}}\right)=\mathrm{log}(\frac{{p}_{t}{r}_{t-1}}{{r}_{t}{p}_{t-1}})$) by means of the following multivariate triangular predictive model (Kostakis et al., 2015):

9 $$\begin{aligned} y_{t} & = \gamma + Ax_{t - 1} + \epsilon_{t} \\ x_{t} & = \rho_{T} x_{t - 1} + \varepsilon_{t} \\ \rho_{T} & = I_{r} + \frac{C}{{T^{\alpha } }} \\ \end{aligned}$$

where $\alpha \ge 0$, $C=\mathrm{diag}({c}_{1},\dots ,{c}_{r})$, $T$ represents the sample size, ${I}_{r}$ is the identity matrix, ${\rho }_{T}$ denotes a matrix of autoregressive coefficients, ${x}_{t}$ is the vector of fundamental variables explaining housing demand and supply, $A$ is a matrix of coefficients, ${\epsilon }_{t}$ and ${\varepsilon }_{t}$ are error terms. It is important to emphasise that various fundamental factors can drive different local housing markets. Therefore, in this study, the set of fundamental factors was selected independently for each city using the leaps-and-bounds algorithm developed by Furnival and Wilson (1974) to maximise the adjusted R^2^ value.^6^ It should also be noted that the fundamental variables ${x}_{t}$ can be integrated of order one, near integrated, mildly stationary, or stationary. Furthermore, since the predictive variables ${x}_{t}$ show a high persistence the standard *t* test under the OLS procedure is invalid for estimating the above model (Cai & Wang, 2014). In addition, the correlation between ${\epsilon }_{t}$ and ${\varepsilon }_{t}$ is usually non-zero (Campbell & Yogo, 2006), leading to an endogenous problem (Stambaugh, 1999). Therefore, the model (9) will be estimated using the IVX method developed by Kostakis et al. (2015), which eliminates these problems by using instrumental variables with a lower degree of persistence than ${x}_{t}$. In particular, this stage involves the construction of mildly stationary instruments as follows:

10 $${\tilde{z }}_{t}={\rho }_{Tz}{\tilde{z }}_{t-1}+{\Delta x}_{t}$$

where ${\Delta x}_{t}={\varepsilon }_{t}+\frac{C}{{T}^{\alpha }}{x}_{t-1}$, ${\rho }_{Tz}={I}_{r}+\frac{{C}_{z}}{{T}^{\beta }}$ with ${C}_{z}=-{I}_{r}$ and $\beta =0.95$. It should also be noted that in the predictive equation in the model (9) there is a constant $\gamma$. In order to obtain the IVX estimator of $A$ that is invariant to the presence of a constant the predictive equation should be transformed by subtracting from it ${\overline{y} }_{t}=\gamma +A{\overline{x} }_{t-1}+{\overline{\epsilon }}_{t}$. As a result of this last action the new predictive equation is given:

11 $${Y}_{t}=A{X}_{t-1}+{\xi }_{t}$$

where ${Y}_{t}={y}_{t}-{\overline{y} }_{t}$, ${X}_{t}={x}_{t}-{\overline{x} }_{t-1}$, ${\xi }_{t}={\epsilon }_{t}-\frac{1}{T}\sum_{t=1}^{T}{\epsilon }_{t}$, ${\overline{y} }_{t}=\frac{1}{T}\sum_{t=1}^{T}{y}_{t}$, ${\overline{x} }_{t-1}=\frac{1}{T}\sum_{t=1}^{T}{x}_{t-1}$, and the IVX estimator of $A$ for the above regression is as follows:

12 $$\widehat{A}=\sum_{t=1}^{T}({y}_{t}-{\overline{y} }_{t}){\tilde{z }}_{t-1}^{^{\prime}}{[\sum_{j=1}^{T}({x}_{j}-{\overline{x} }_{t-1}){\tilde{z }}_{j-1}^{^{\prime}}]}^{-1}$$

For an empirical estimation of the parameters of the model (9), the IVX R package will be used in this study.

Then, in order to decompose the log price-to-rent ratio ($\mathrm{log}{p}_{t}-\mathrm{log}{r}_{t}$) into fundamental (${f}_{t}$) and non-fundamental (${nf}_{t}$) components, one should determine the fitted values of the growth rates of the price-to-rent ratio as ${\widehat{y}}_{t}={\widehat{Y}}_{t}+{\overline{y} }_{t}$. Then, in a second stage, the values of the above-mentioned elements (${f}_{t}$ and ${nf}_{t}$) in each period can be calculated using formulas:

13 $${\widehat{f}}_{t}={\widehat{f}}_{1}+\sum_{i=2}^{t}{\widehat{y}}_{i}$$

14 $${nf}_{t}=\mathrm{log}{p}_{t}-\mathrm{log}{r}_{t}-{\widehat{f}}_{t}$$

where ${\widehat{f}}_{1}=\mathrm{log}{p}_{1}-\mathrm{log}{r}_{1}$. Confirmation that Eq. (13) allows for the calculation of the fundamental component of the log price-to-rent ratio can be presented as follows. Firstly, the second element on the right-hand side of Eq. (13), i.e., $\sum_{i=2}^{t}{y}_{i}$ can be written as:

15 $$\sum_{i=2}^{t}{y}_{i}=\mathrm{log}\left(\frac{{p}_{2}{r}_{1}}{{r}_{2}{p}_{1}}*\frac{{p}_{3}{r}_{2}}{{r}_{3}{p}_{2}}*\frac{{p}_{4}{r}_{3}}{{r}_{4}{p}_{3}}*\dots *\frac{{p}_{t}{r}_{t-1}}{{r}_{t}{p}_{t-1}}\right)=\mathrm{log}\left(\frac{{r}_{1}{p}_{t}}{{p}_{1}{r}_{t}}\right)=\mathrm{log}\left(\frac{{p}_{t}}{{r}_{t}}\right)-\mathrm{log}\left(\frac{{p}_{1}}{{r}_{1}}\right)$$

When comparing Eqs. (13) and (15), one can see that ${f}_{t}=\mathrm{log}\left(\frac{{p}_{t}}{{r}_{t}}\right)$ and ${\widehat{f}}_{t}=\widehat{\mathrm{log}}\left(\frac{{p}_{t}}{{r}_{t}}\right)$. In turn, Eq. (14) allowing for the calculation of the non-fundamental component results directly from the assumption made in Eq. (4).

### Price bubble detection method

It should be noted that the non-fundamental component can exhibit large downward movements, explosive behaviour and also follows a random process. If there is only a process of random walk in a given time series (allowing for a small trend), this means that the market is operating under normal conditions. In order to investigate this phenomenon, the PSY procedure can be used, for which the zero hypothesis of no bubble or crisis is as follows:

16 $${H}_{0}: {nf}_{t}=k{T}^{-\varphi }+{nf}_{t-1}+{\mu }_{t}$$

where ${\mu }_{t}$ denotes the error term, $k{T}^{-\varphi }$ is the intercept with $k$ and $\varphi >0.5$ enabling for drift in the data, which is justified in the case of a study of the log price-to-rent ratio in the housing market. However, alternative hypotheses can be constructed as follows:

17 $${H}_{A}: {nf}_{t}=\left(1+k{T}^{-\eta }\right){nf}_{t-1}+{\mu }_{t} \,\mathrm{for\,explosive\,behaviour}$$

18 $${H}_{A}: {nf}_{t}=-{L}_{t}+{nf}_{t-1}+{\mu }_{t} \,\mathrm{for\,downward\,behaviour}$$

where $k>0$, $\eta \in (\mathrm{0,1})$, ${L}_{t}$ denotes a random sequence. Bearing in mind that the PSY procedure is a right-sided test, it is important to underline the fact that it also unexpectedly enables the detection of collapse behaviour, as emphasised by Phillips and Shi (2019). In order to empirically verify the above hypotheses for each observation $r$ in the time series, the PSY method uses the BSADF statistic, which can be calculated as:

19 $${BSADF}_{r}: \underset{}{\mathrm{max}}\left\{{ADF}_{{r}_{1}}^{{r}_{2}}:{r}_{2}=r \,\mathrm{and}\, {r}_{1}\in [1,r-{r}_{0}+1]\right\}$$

where ${ADF}_{{r}_{1}}^{{r}_{2}}$ denotes the ADF statistic calculated for a subsample of $T$ from observation ${r}_{1}$ to ${r}_{2}$, ${r}_{0}$ represents the size of the minimum window which is required to run the ADF regression that in this study takes the form:

20 $$\Delta {nf}_{t}={\beta }_{0}+{\beta }_{1}{nf}_{t-1}+\sum_{i=1}^{L}{\omega }_{i}\Delta {nf}_{t-1}+{\mu }_{t}$$

where $L$ is the optimum lag order selected on the BIC criteria, ${\beta }_{0}$, ${\beta }_{1}$, ${\omega }_{i}$ are coefficients of the model and $t\in [{r}_{1},\dots ,{r}_{2}]$. It should be noted that the ADF statistic corresponds to the t-statistic estimated for parameter ${\beta }_{1}$ by using the OLS method. In this study the maximum lag order equal to 3 has been selected and ${r}_{0}$ has been set following Phillips et al. (2015a) as ${r}_{0}=T(0.01+\frac{1.8}{\sqrt{T}})$. It should also be noted that, using the framework of Eq. (20), hypotheses (16)–(19) can be written in the following form:

21 $${H}_{0}: {\beta }_{0}=k{T}^{-\varphi }\, \mathrm{and} \,{\beta }_{1}=0$$

22 $${H}_{A,bubble}: {\beta }_{0}=0\, \mathrm{and}\, {\beta }_{1}>0$$

23 $${H}_{A,collapse}: {\beta }_{0}=-E\left({L}_{t}\right)\, \mathrm{and}\, {\beta }_{1}=0$$

Then, in order to check whether the observation $r$ is from the bubble process, it is necessary to verify whether the corresponding BSADF statistic is greater than the critical value. The latter can be obtained by means of the Monte Carlo simulation, which is implemented in the psymonitor R package developed by Phillips and Shi (2020) as well as in the MultipleBubbles R package. This analysis has used 2000 repetitions to determine the critical value.

## Results and discussion

### IVX and OLS estimates

In the first stage of the empirical study, the predictive equation from the model (9) was estimated using both IVX and OLS methods. The results of this analysis are presented in Table 2. The first conclusion that can be drawn from the estimation is that there is a very large consensus in the parameter estimates between the two applied techniques. In particular, the differences between IVX and OLS results are almost imperceptible both in terms of the magnitude of individual parameters and their significance. These can only be observed for the cities of Katowice and Wroclaw. In the former case, the difference lies in the lack of significance of the parameter in the dynamic consumption index variable when estimating with the IVX procedure. In the latter case, i.e., for Wroclaw, the difference is identical but only in terms of the log new housing stock variable. When comparing the adjusted ${R}^{2}$ values, there are also no significant dissimilarities for particular estimation methods. In general, they range from 0.20 to 0.55, indicating good matching of the models to the data compared to other studies (see, e.g., Hwang & Quigley, 2006; Al-Masum & Lee, 2019). Furthermore, as Stevenson (2008) notes, not obtaining very high R^2^ values in studies regarding the occurrence of house price bubbles is not worrisome because the purpose of this type of analysis is not to explain actually existing market movements but to model fundamental house prices.

**Table 2:** Estimated coefficients for the predictive regression using the IVX and OLS methods

City	Regressors								Method	Adj. R^2^
	Log new housing stock	Log monthly gross salary	Log number of unemployed	Real interest rate	Log real money supply	Log real new household borrowing	Dynamic consumption index	Log real annual housing rent		
Katowice	NA	NA	0.26***	NA	NA	NA	0.01	0.98***	IVX	0.40
	NA	NA	0.26***	NA	NA	NA	0.01**	0.91***	OLS	0.36
Bydgoszcz	NA	NA	0.25***	− 0.01*	NA	NA	NA	0.62***	IVX	0.26
	NA	NA	0.24***	− 0.01*	NA	NA	NA	0.63***	OLS	0.26
Kielce	NA	0.40**	NA	0.02	NA	NA	− 0.01	0.52***	IVX	0.20
	NA	0.42**	NA	0.02	NA	NA	− 0.01	0.54***	OLS	0.21
Cracow	0.06	− 0.82*	0.63***	− 0.01	0.64**	0.17***	0.02***	1.14***	IVX	0.55
	0.06	− 0.82*	0.63***	− 0.01	0.64**	0.17**	0.02***	1.15***	OLS	0.55
Lublin	− 0.08	0.67**	0.69***	NA	NA	0.32*	0.01	0.70***	IVX	0.31
	− 0.09	0.72**	0.69***	NA	NA	0.33**	0.01	0.71***	OLS	0.32
Lodz	NA	− 0.41	0.17	NA	NA	− 0.12*	NA	0.75***	IVX	0.27
	NA	− 0.41	0.17	NA	NA	− 0.12*	NA	0.76***	OLS	0.28
Olsztyn	NA	− 0.38	NA	NA	0.32**	NA	NA	0.95***	IVX	0.53
	NA	NA	NA	NA	0.33**	NA	NA	0.95***	OLS	0.53
Poznan	0.05*	NA	0.30***	NA	NA	− 0.09*	0.01*	0.80***	IVX	0.24
	0.05*	NA	0.30***	NA	NA	− 0.10**	0.01**	0.81***	OLS	0.24
Rzeszow	NA	0.69*	0.37**	NA	− 0.42	NA	0.01*	0.73***	IVX	0.38
	NA	0.68*	0.37**	NA	− 0.42	NA	0.01**	0.74***	OLS	0.38
Szczecin	NA	NA	0.25***	NA	0.25***	NA	0.01	0.92***	IVX	0.45
	NA	NA	0.25***	NA	0.26**	NA	0.01	0.92***	OLS	0.45
Warsaw	NA	0.20**	0.22***	− 0.01***	NA	NA	NA	0.78***	IVX	0.20
	NA	0.21**	0.22***	− 0.01***	NA	NA	NA	0.77***	OLS	0.21
Gdansk	NA	− 0.26*	0.32***	NA	NA	NA	0.01	0.78***	IVX	0.50
	NA	− 0.28**	0.32***	NA	NA	NA	0.01	0.79***	OLS	0.50
Wroclaw	0.07	NA	0.17*	NA	NA	− 0.05	− 0.01	0.51***	IVX	0.23
	0.06*	NA	0.17***	NA	NA	− 0.06	− 0.01	0.52***	OLS	0.24

***One per cent level of significance; **five per cent level of significance; *ten per cent level of significance. NA means not applicable

**Fig. 6:**
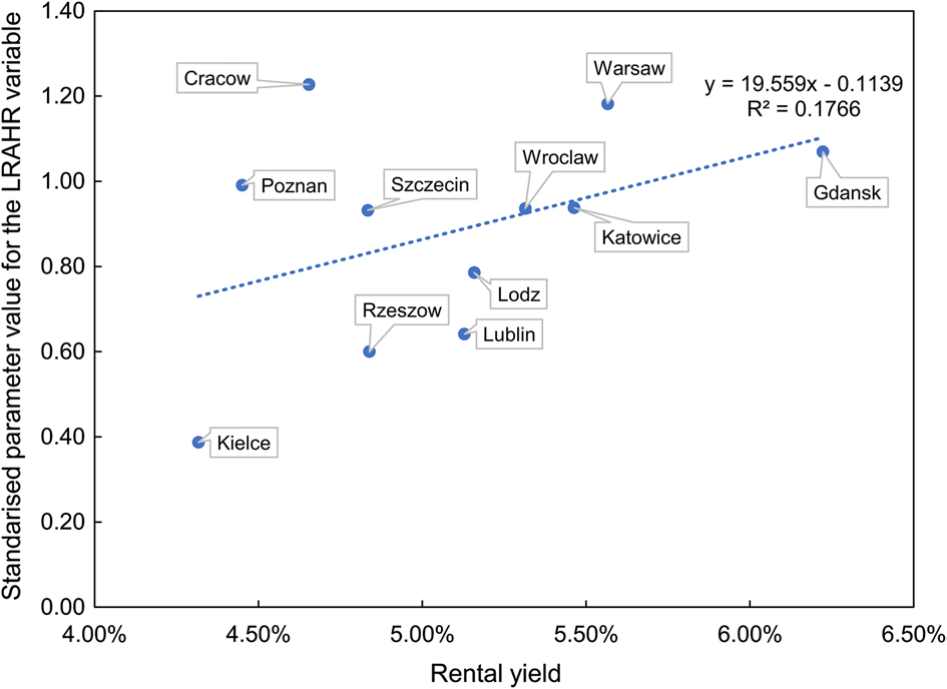
The relationship between parameter values for the LRAHR variable and rental yields in studied cities. *Note* LRAHR means log real annual housing rent. Data on rental yields were obtained from studies carried out by Marona et al. (2020), and they include the average values from 2016 to 2019. Due to a lack of data on rental yields for Bydgoszcz and Olsztyn, results for these cities are not presented in the figure

When looking in detail at the significance of individual parameters, attention should be paid to the covariate describing the log real housing rent, which turned out to be significant for all the examined cities. It should be noted that for this variable, the parameter is positive, which implies that an increase in housing rent in a given quarter leads to an increase in the dependent variable in the next period. The mechanism of this impact can be explained as follows. Investors observing an increase in residential rents in period $t$ may intensify the demand for residential real estate for rent in period $t+1$, which in turn may lead to excessive price rises, which at the same time increase the price-to-rent ratio. It should also be noted that the strength of the parameter when discussing the variable is closely correlated with the rental profitability in a given city. As shown in Fig. 6, it is a positive dependence, i.e., the higher the rental yield, the stronger the magnitude of the standardised parameter at the log real housing rent variable. This is because, in more profitable (for investors) residential markets, the level of rent is relatively high compared to the price level, which also means a low price-to-rent ratio. Therefore, as noted above, a rise in rents in the current period results in price growth in the next period, leading to significant growth in the price-to-rent ratio.

Another regressor that has proved to be a significant predictor of the price-to-rent ratio growth rate in most of the analysed cities concerns the log number of unemployed. Again, the positive impact on the dependent variable can be considered here, but to a much lesser degree than in the case of the log real housing rent variable. In particular, a 1% growth in the number of unemployed increases the dependent variable of about 0.17–0.69% depending on the specific city. On this basis, it can be concluded that the rise in unemployment over a given period of time implies a greater fall in rents than prices in the next quarter, leading to an increase in the price-to-rent ratio. It should be noted that the strong impact of the level of unemployment on the level of prices and rents in the Polish housing market has already been repeatedly confirmed by other studies carried out by Tomal (2019a, b) and Leszczyński and Olszewski (2017), among others.

The other covariates have been found to be significant only occasionally. For example, the real interest rate variable is a relevant predictor of the growth rate of the price-to-rent ratio in Bydgoszcz and Warsaw. The relationship observed is negative, i.e., an increase in the real interest rate leads to a decrease in the dependent variable. This situation can be explained as follows. A rise in the interest rate determines, on the one hand, an increase in the attractiveness of bank deposits and, on the other hand, a reduction in investments due to a higher cost of credit. All this leads to a decline in demand for residential properties and, consequently, a fall in their prices. With respect to rent levels, elevated interest rates may cause them to rise as a result of higher borrowing costs and landlords' desire to pass them on to tenants.

Furthermore, the parameters at covariates describing log real new household borrowing and log monthly gross salary have different directions of influence on the dependent variable between cities. However, it should be noted that such ambiguous and even theory-defying results have already been confirmed several times in other studies that analysed the impact of macrodeterminants on housing prices in Poland (i.e., Posedel & Vizek, 2009; Tomal, 2019a). On the other hand, a high degree of agreement in the direction of the impact is present for the dynamic consumption index and the log real money supply regressors. To the best of the author's knowledge, there has been no research in Poland analysing the impact of the above-mentioned variables on the price level in the housing market. However, the positive correlation obtained in this study seems to be consistent with the theory. In particular, following the increase in money supply, larger amounts of money flow into the housing market, which contributes to maintaining the upward price trend (Su et al., 2019), increasing the price-to-rent ratio. In the context of consumption levels, Attanasio et al. (2009) emphasise that house price and consumption growth have been closely synchronised over the past several decades. Moreover, high consumption reflects high demand, including for housing goods, which may also lead to their higher prices. Finally, the last covariate concerned log new housing stock and proved insignificant for most of the housing markets surveyed as also confirmed by Leszczynski and Olszewski (2017).

### Construction of non-fundamental components and bubble detection

**Fig. 7:**
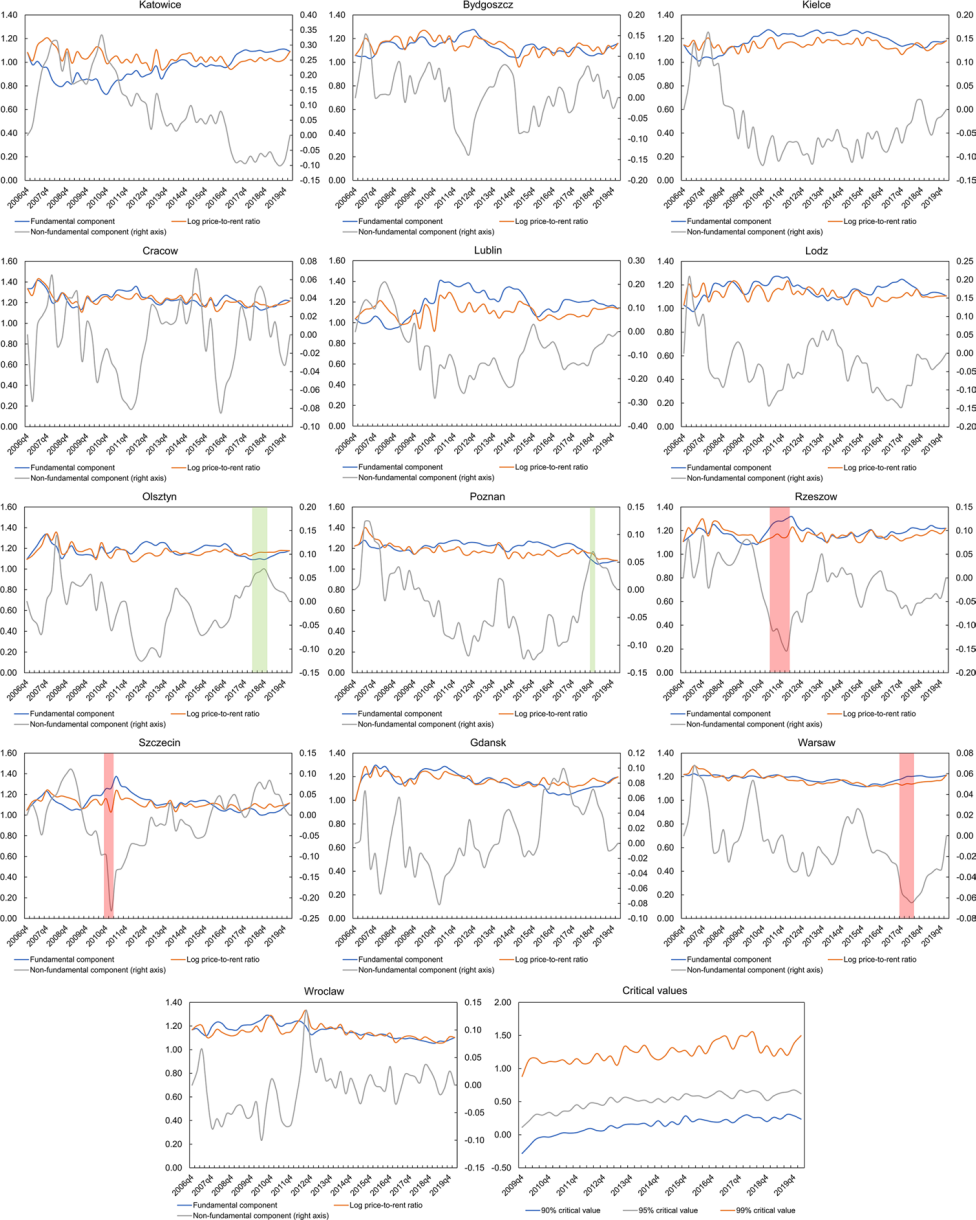
Identified explosive (green bars) and downward movements (red bars) of the non-fundamental components of the log price-to-rent ratios in Polish major housing markets. *Note* This figure refers to calculations obtained from the IVX method. In the case of the log price-to-rent ratio decomposition based on the OLS method, the periods identified by the PSY test are almost identical. For Olsztyn and Poznan, Explosive movements appeared in 2018q2–2018q4 and 2018q4, respectively. For Rzeszow, Szczecin, and Warsaw, downward movements appeared in 2011q2–2012q1, 2010q4–2011q1 and 2017q4–2018q2, respectively. (Color figure online)

In the next stage of the study, the log price-to-rent ratios into fundamental and non-fundamental components were decomposed using Eqs. (13) and (14) for each city. Then, for the latter, the PSY test was used to detect price bubbles in the markets studied. The results of this analysis are illustrated in Fig. 7, showing identified explosive (green bars) and downward movements (red bars) of the non-fundamental components over the period 2009q4–2020q1 (minimum window size was 12 observations). On its basis, it can be concluded that the non-standard behaviour of the non-fundamental component in individual cities was usually short, i.e., lasting up to four quarters.

By analysing the housing markets studied in detail, extremely interesting conclusions can be drawn. First of all, there are explosive behaviours of the non-fundamental component in some cities, as can be seen in Olsztyn in 2018q2–2018q4 and Poznan in 2018q4. It is also worth noting the behaviour of the log price-to-rent ratio after the bubble burst. In particular, not in every case, the value of this relation decreases simultaneously with the value of the non-fundamental component. This type of situation can be observed, among others, in Olsztyn, where the 2018q4 climax is followed by a drastic decrease in the value of the residual component, but the log price-to-rent ratio in the next period increases due to the growth of the fundamental component. Therefore, in this case, based solely on an analysis of the log price-to-rent ratios, wrong conclusions could be drawn as regards the identification of housing market bubbles.

In turn, Rzeszow, Szczecin and Warsaw, show downward movements of the non-fundamental component of the log price-to-rent ratio. In the cases of the first two cities, this situation occurred between 2010 and 2012, i.e., during a period of prolonged price decline following the 2007–2008 financial crisis, which lasted more or less until 2013 (see Figs. 1, 2, 3). However, in the analysed cases, one cannot speak of the occurrence of a crisis as such, but rather of the phenomenon of a negative price bubble. The latter exist when observed in the market the log price-to-rent ratios are below their equilibrium values (Brzezicka, 2020; Teng et al., 2013)**.** As Shiller (2003) points out, the phenomenon of a negative price bubble can be explained very similarly to its positive version. Namely, the downward movements in real estate prices/price-to-rent ratios do not correspond to the economic fundamentals, but rather are the result of very pessimistic prognoses of investors. This explanation also applies to the situation on residential markets in Rzeszow and Szczecin. Namely, as shown in Fig. 7, in 2010–2012, there was an increase in the fundamental components of log price-to-rent ratios. However, their actual values remained stable or slightly decreased, which could be a result of the pessimistic atmosphere prevailing in the Polish residential market at that time.

On the other hand, in terms of the Warsaw residential market, a negative price bubble occurred between 2017q4 and 2018q2. This finding is confirmed by the data of the National Bank of Poland (2020), according to which the housing market in Warsaw has been in an expansion phase since 2015, but a change in its behaviour can be observed in 2017–2018. In particular, the number of flats sold decreased, developers reduced the scale of their residential projects and the number of flats offered on the market. Finally, looking at Fig. 7, one more conclusion can be drawn. Namely, analysing the behaviour of the non-fundamental component in all surveyed cities, one may notice that, more or less at the turn of 2013 and 2014, its dynamics reversed, i.e., its values started to grow, which corresponds to changes in the level of housing prices presented in Figs. 1, 2 and 3. However, the scale of this growth was not so large for individual markets that one could speak of a price bubble.

**Fig. 8:**
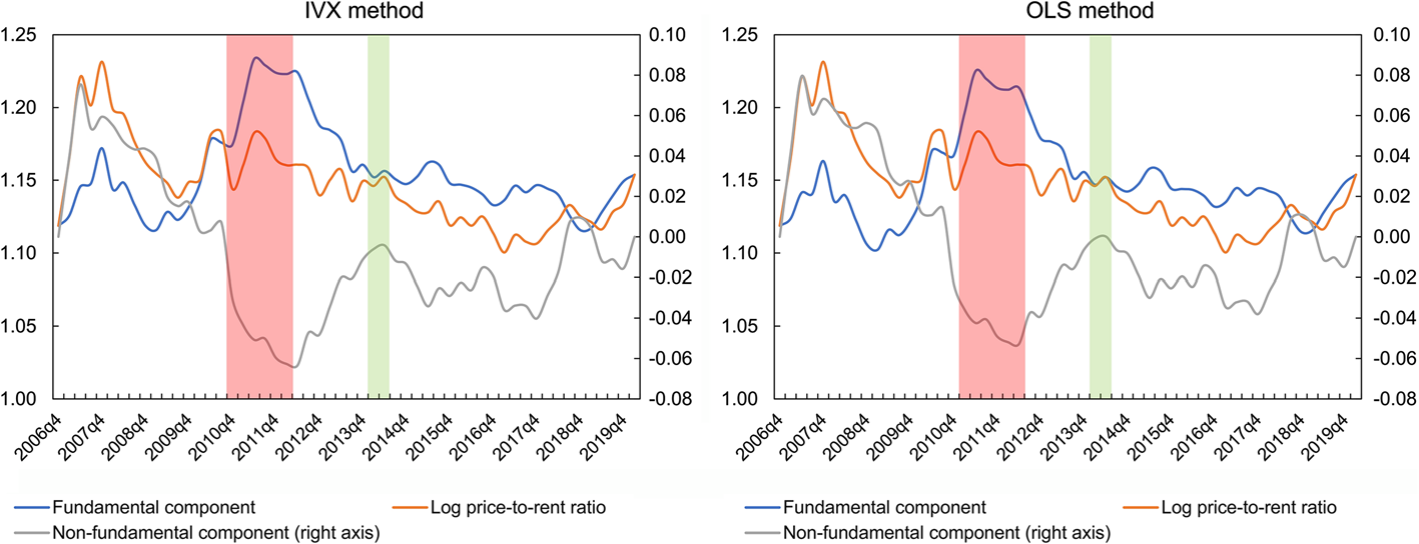
Average values of the fundamental and non-fundamental components of the log-price-to-rent ratios. *Note* Explosive movements (green bars) appeared in 2014q1–2014q2. Downward movements (red bars) appeared in 2010q4–2012q1. (Color figure online)

**Fig. 9:**
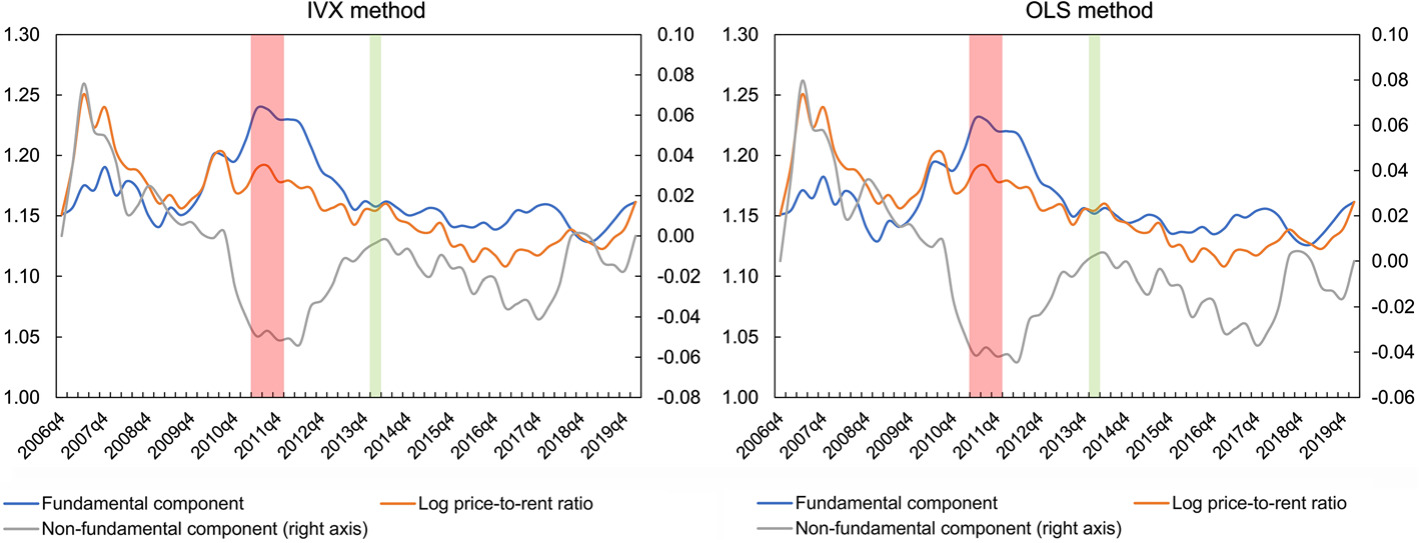
Weighted average values of the fundamental and non-fundamental components of the log-price-to-rent ratios. *Note* As a weight, the total population was used. Explosive movements (green bars) appeared in 2014q1. Downward movements (red bars) appeared in 2011q2–2011q4. (Color figure online)

In order to analyse thoroughly the behaviour of the housing market as a whole, average values of the log price-to-rent ratio and its components were calculated for the examined cities. The results of this analysis are presented in Fig. 8. Additionally, in order to check the obtained results, the same time series are presented in Fig. 9, but calculated using a weighted average, where the population level in a given city is taken as a weight. On the basis of the obtained results, it can be seen that regardless of the model estimation method, approximately between 2010 and 2012 there was a strong negative price bubble in the Polish housing market, and then the non-fundamental component started to grow. It should be noted that the above conclusions fit very well with the general dynamics of the Polish real estate market. In particular, according to research conducted by the National Bank of Poland (2020), this market, after a period of cooling caused by the 2007–2008 financial crisis, has been in the recovery phase between 2013 and 2015. The change in the behaviour of the Polish housing market in the period 2013–2015 is also validated by other studies on price convergence. Specifically, research carried out by Tomal (2019a) show that since the above-mentioned period, there has been a process of price divergence across regional housing markets following a period of very strong convergence. This divergence dynamic may be due to the intensification in speculative activity in individual housing markets, leading to excessive price increases in some cities.

When referring the present research results to others addressing the issue of identification of bubbles in the Polish housing market, one can observe both similarities and differences. In particular, Żelazowski (2018), who investigated the price-to-income indicator between 2003 and 2016, did not identify a positive bubble in any Polish major residential market after 2008, conversely to the present analysis, which may be due to the lack of consideration given to housing market fundamentals during the analysis. However, some common ground can also be found between the study done by Żelazowski (2018) and this survey. Namely, the latter Author found a downward phase of the housing market in some Polish provincial cities between 2011 and 2013, which is consistent with the results of this survey. It should also be noted that this analysis is also in line with the research done by Czerniak and Kawalec (2020), who found that after 2016 there is no indication that there was a price bubble in the Polish housing market.

### Robustness test

The multivariate triangular predictive model outlined by Kostakis et al. (2015), which is used in this study, does not account for serial correlation and heteroscedasticity in the error term of the linear predictive regression model. Yang et al. (2020) demonstrated that when autocorrelation of residuals is present, the latter model exhibits severe size distortions. Therefore, Yang et al. (2020) proposed a correction to the model presented in Eq. (9), which assumes that the error term ${\epsilon }_{t}$ follows an AR(q) + GARCH(m, n) process:

$$\begin{aligned} \epsilon_{t} & = \theta_{1} \epsilon_{t - 1} + \cdots + \theta_{q} \epsilon_{t - q} + v_{t} \\ v_{t} & = h_{t} e_{t} \\ h_{t}^{2} & = \omega + \mathop \sum \limits_{i = 1}^{m} a_{i} v_{t - i}^{2} + \mathop \sum \limits_{j = 1}^{n} b_{j} h_{t - j}^{2} \\ \end{aligned}$$

where ${\theta }_{1},\dots ,{\theta }_{q}$ are the coefficient for the AR(q).

Taking the above into account, for each analysed city, it was checked whether the residuals from model (9) are characterised by serial correlation using the Ljung-Box test. A lag number of 3 was chosen for the test, which is optimal for time series having about 50 observations (Hassani and Yeganegi, 2020). The results of the autocorrelation residual analysis are presented in Table 3, from which it can be concluded that this problem occurs only for three cities, i.e., Kielce, Rzeszow and Gdansk. Therefore, the entire study was conducted anew for these cities but assuming model (9) estimation based on the approach presented by Yang et al. (2020). In particular, the empirical specification assumed that ${\epsilon }_{t}$ is governed by an AR(1) + GARCH(1, 1) process. Taking a higher value of q for the AR process would probably increase the model's performance, but on the other hand, it reduces the number of observations, which from the point of view of study based on short time series is a drawback of this method.

**Table 3:** Ljung–Box test results for ${\epsilon }_{t}$ from model (9)

City	Lag		
	1	2	3
Katowice	0.71	2.19	2.48
Bydgoszcz	0.45	0.62	2.81
Kielce	5.81***	7.55***	7.68***
Cracow	1.83	3.62	5.66
Lublin	1.13	1.56	2.70
Lodz	0.72	0.88	0.92
Olsztyn	0.05	0.55	1.16
Poznan	0.46	0.54	0.63
Rzeszow	1.76	1.90	7.38**
Szczecin	0.79	1.60	2.38
Gdansk	2.89**	4.99**	11.29***
Warsaw	1.37	2.03	2.19
Wroclaw	0.37	3.55	4.02

***One per cent level of significance; **five per cent level of significance

Table 4 contains the summary results of the robustness test, particularly the parameter estimates of model (9) and the results provided by the PSY procedure. It should be noted that new results almost completely coincide with those obtained on the basis of model (9) estimated with the algorithm proposed by Kostakis et al. (2015). The only difference concerns the city of Gdansk, where, contrary to the baseline procedure, the one based on the Yang et al. (2020) model indicated that there was a crisis in this housing market in the third quarter of 2019. In conclusion, the conducted robustness test confirmed the reliability of the previously obtained estimates.

**Table 4:** Estimated coefficients for the predictive regression using the Yang et al. (2020) model

Analysis	City		
	Kielce	Rzeszow	Gdansk
Yang et al. (2020)* model estimates*			
Log real annual housing rent	0.32***	0.45***	0.68***
Real interest rate	0.01**	NA	NA
Dynamic consumption index	− 0.01	0.01	0.01
Log monthly gross salary	0.28**	0.88**	− 0.21
Log number of unemployed	NA	0.26**	0.28***
Log real money supply	NA	− 0.44	NA
Ljung–Box test (lag = 3)	1.41	3.88	5.60
Adjusted R^2^	0.14	0.33	0.37
*PSY procedure*			
Explosive movements	NA	NA	NA
Downward movements	NA	2011q2–2012q1	2019q3

***One per cent level of significance; **five per cent level of significance; *ten per cent level of significance. NA means not applicable

## Conclusions

### Findings

On the basis of the studies carried out, several main conclusions can be drawn:

1. Comparing the estimation results of the model (9) using IVX and OLS methods, there are no significant differences, which indicates that standard multiple regression is a fairly good tool for the decomposition of the log price-to-rent ratio.
2. In the analysed period, both explosive and downward movements of the non-fundamental component of the log price-to-rent ratio could be observed in housing markets operating in Polish voivodship cities. However, these extraordinary movements lasted quite shortly from one to a maximum of four quarters.
3. Looking at the Polish residential market as a whole throughout 2011, the log price-to-rent ratios in the examined cities were below their equilibrium values shaped by market fundamentals. As a result, there was a so-called negative price bubble during this period. In turn, at the begging of 2014, explosive movements of the non-fundamental component of the log price-to-rent ratio appeared.
4. Comparing the results obtained with other studies on the behaviour of the Polish housing market, there are quite significant differences, which may be due to the lack of consideration of the housing market fundamentals in previous analyses.

### Study limitations and future research directions

This study has several limitations. The first, and the main one, is the relatively short analysis period due to the lack of availability of housing data before 2006q3. Another research constraint was the absence of information on certain housing market fundamentals that shape residential supply and demand. In particular, this concerned, among others, the real interest rate (the only data are available at the national level) and other variables, such as demographics (the only data available are semi-annual). The orientations for future research are directly related to the limitations set out. Specifically, future studies should take into account longer time series in order to confirm the results obtained in this article and exclude the possibility of the emergence of accidental blips during the PSY procedure (Phillips & Shi, 2018). It should also be noted that, to the best of the author's knowledge, this paper is the first one in which the identification of price bubbles in the Polish housing markets has been made after a previous decomposition of the log price-to-rent ratio. Therefore, future research may confront the results obtained in this study with those obtained under other methods of disaggregation of the log price-to-rent ratio. Furthermore, the PSY procedure used in this study cannot provide an answer to whether the non-fundamental component of the log price-to-rent ratio will change or remain stable in the future. Therefore, the next research should focus on a generative mechanism for the bubble with driver variables serving as predictors (Greenaway-McGrevy & Phillips, 2016).

### Implications for practice and policy

This study has implications for both practice and policy. Specifically, legislators, when they have information that price increases in the housing market are unjustified by fundamental economic factors, should apply appropriate monetary and/or fiscal policies to reduce the magnitude of inflation, which will also allow avoiding a possible economic downturn following the bursting of the bubble. On the other hand, when market fundamentals justify rises in the housing market, the government should not intervene because, as Fraser et al. (2008) note, this could result in a misallocation of resources. Moreover, investors and households need to know whether current prices on the housing market correspond to their fundamental values. The former may assess the level of risk connected with a given investment, while the latter may make more rational decisions regarding the purchase or rental of residential real estate. Referring to the above implications to the Polish residential market, it should be pointed out that currently, despite the high growth dynamics in this market, there are no reasons for the government to take legislative actions, as the fundamental factors justify the dynamics. These are also important findings for housing investors for whom the Polish residential market is presently a safe place to invest. In the context of Polish households, it is important to note that they should not postpone their decisions to buy or rent a flat, as the likelihood of significant price reductions resulting from a bursting bubble is minimal. It also appears that the above implications are also valid in the era of the COVID-19 pandemic. In particular, the upward dynamics of sales prices continues as indicated by National Bank of Poland (2020) data. On the other hand, rental prices fell by up to 10% in major Polish cities as a result of the introduction of lockdowns, which increased unemployment (Tomal & Marona, 2021) and the redirecting of a large part of the short-term rental supply to long-term rentals (Trojanek et al., 2021). However, it can be predicted with high probability that the situation in the Polish rental market has already returned to normal or will return in the near future due to significant loosening COVID-19 restrictions, dynamically advancing vaccination and the fact that the population is accustomed to the presence of coronavirus in everyday life in Poland.

## Footnotes

1 Non-fundamental factors, on the other hand, may include noise, feedback trading, or irrational expectations, among others (Lee, 1998).

2 In the context of research on price bubbles, the Polish residential market should be understood as the market of sales and rentals of flats, excluding other types of residential properties as well as residential land. This results from the fact that in Poland, data on the level of average prices in the housing market is available only for flats.

3 Actual housing prices are those currently observable in the market. In contrast, their fundamental counterparts reflect the behavior of housing market fundamentals. Finally, the value of non-fundamental component is the difference between the actual and fundamental price.

4 For example, analysing the current (as of 12.08.2021) ads for flats for rent in Warsaw from the largest Polish online portal otodom.pl, one can see that a maximum of 5% of them come from the primary market, i.e. they were purchased, finished, and intended for rental.

5 Although the data are for the period 2006q3–2020q1 potential bubble behaviour is identified as early as 2009q4–2020q1. This is because a total of 13 observations are discarded during model (9) calibration and the PSY test.

6 This stage extends the housing bubble detection procedure proposed by Shi and Phillips (2020) and its goal is to obtain more accurate estimates of fundamental house prices.
